# Surgical Cleanliness

**Published:** 1896-02

**Authors:** 


Surgical Cleanliness; or, Asepsis Explained, is the title of a new book which will shortly be published by Dr. L. D. Rogers, editor of *The People's Health Journal* of Chicago. It is designed for Physicians, Students, and Nurses.

